# Uncovering Neuronal Networks Defined by Consistent Between-Neuron Spike Timing from Neuronal Spike Recordings

**DOI:** 10.1523/ENEURO.0379-17.2018

**Published:** 2018-05-21

**Authors:** Roemer van der Meij, Bradley Voytek

**Affiliations:** 1 Department of Cognitive Science, University of California, San Diego, La Jolla, CA 92093; 2 Neurosciences Graduate Program, University of California, San Diego, La Jolla, CA 92093; 3 Halicioglu Data Science Institute, University of California, San Diego, La Jolla, CA 92093; 4 Kavli Institute for Brain and Mind, University of California, San Diego, La Jolla, CA 92093

**Keywords:** action potentials, decomposition, neuronal networks, neuronal spiking, spike timing

## Abstract

It is widely assumed that distributed neuronal networks are fundamental to the functioning of the brain. Consistent spike timing between neurons is thought to be one of the key principles for the formation of these networks. This can involve synchronous spiking or spiking with time delays, forming spike sequences when the order of spiking is consistent. Finding networks defined by their sequence of time-shifted spikes, denoted here as spike timing networks, is a tremendous challenge. As neurons can participate in multiple spike sequences at multiple between-spike time delays, the possible complexity of networks is prohibitively large. We present a novel approach that is capable of (1) extracting spike timing networks regardless of their sequence complexity, and (2) that describes their spiking sequences with high temporal precision. We achieve this by decomposing frequency-transformed neuronal spiking into separate networks, characterizing each network’s spike sequence by a time delay per neuron, forming a spike sequence timeline. These networks provide a detailed template for an investigation of the experimental relevance of their spike sequences. Using simulated spike timing networks, we show network extraction is robust to spiking noise, spike timing jitter, and partial occurrences of the involved spike sequences. Using rat multineuron recordings, we demonstrate the approach is capable of revealing real spike timing networks with sub-millisecond temporal precision. By uncovering spike timing networks, the prevalence, structure, and function of complex spike sequences can be investigated in greater detail, allowing us to gain a better understanding of their role in neuronal functioning.

## Significance Statement

Spike timing consistencies in neuronal networks are widely thought to be one of several key principles behind neuronal functioning. They are challenging to investigate, however, because there is effectively an infinite number of combinations of neurons and their between-neuron time delays for any given recording. Many techniques have been developed for their analysis, but they are still limited by the complexity of spike timing patterns they can reveal. Here, we present a novel approach that can reveal spike timing patterns with arbitrary combinatorial complexity. This provides a new opportunity for investigating spike timing networks, which is crucial to gain a better understanding of the role they play in neuronal functioning.

## Introduction

Distributed networks of neurons, or cell assemblies, are widely assumed to be fundamental to brain functioning (Hebb, 1949; Treisman, 1996; Singer, 1999; Varela et al., 2001; Harris, 2005; Buzsáki, 2010). A subset of these networks is thought to be formed by consistent timing of action potentials, or spikes, between neurons (Bienenstock, 1995; Singer, 1999; Ainsworth et al., 2012; Feldman, 2012), a feature of spike recordings across species (Mainen and Sejnowski, 1995; Salinas and Sejnowski, 2001; VanRullen et al., 2005; Fujisawa et al., 2008; Ratté et al., 2013). The spiking between neurons of such networks can be synchronous or involve time delays (Izhikevich, 2006; Fujisawa et al., 2008; Sakurai et al., 2013), forming spike sequences when firing in a consistent order (Lee and Wilson, 2002; Ikegaya et al., 2004; Tiesinga et al., 2008). Spike sequences can involve the same neurons and occur within the same time window (Mao et al., 2001; MacLean et al., 2005; Luczak et al., 2007; Matsumoto et al., 2013; Miller et al., 2014). Although there is still much debate about how important spike timing is in comparison to alternatives such as rate-based coding schemes (Kumar et al., 2010; Rolls and Treves, 2011; Ainsworth et al., 2012), the investigation of spike timing networks and their spike sequences remains necessary to further our understanding of basic neuronal operations.

Finding networks defined by their sequences of consistent time-shifted spikes between neurons, denoted in the following as spike timing networks, is a tremendous challenge due to their possible complexity, as neurons can participate in multiple spike sequences at a continuum of between-spike time delays. The past decades have seen the arrival of many methods that can characterize spike timing networks (Abeles and Gerstein, 1988; Chapin and Nicolelis, 1999; Nádasdy et al., 1999; Tetko and Villa, 2001; Grün et al., 2002; Lee and Wilson, 2002; Schnitzer and Meister, 2003; Ikegaya et al., 2004; Okatan et al., 2005; Schneider et al., 2006; Nikolíc, 2007; Pipa et al., 2008; Schrader et al., 2008; Berger et al., 2010; Eldawlatly et al., 2010; Louis et al., 2010; Peyrache et al., 2010; Humphries, 2011; Lopes-dos-Santos et al., 2011; Gansel and Singer, 2012; Torre et al., 2016). Their application has led to important insights, yet they have several limitations, especially when it comes to their application on large scale neuronal recordings (Buzsáki, 2004). Namely, either: (1) the complexity of the identified networks is limited due to combinatorial explosion with increasing network size (e.g., template searching); (2) the networks are described only by the association of their member neurons without describing spike sequences; (3) between-spike time delays >0 are either discarded or not recovered; (4) temporal binning of spike times leads to reduced temporal precision; (5) networks with overlapping member neurons are not separated; or (6) a combination of the above. Although not important for every investigation of interactions in spiking networks (e.g., for higher order interactions see, Nakahara and Amari, 2002; Yu et al., 2009; Eldawlatly et al., 2010; Staude et al., 2010; Balaguer-Ballester et al., 2011; Shimazaki et al., 2012), they are essential for the exact identification of neurons and their spike sequences, and investigating their occurrence as a function of experimental variables.

**Figure 1.**
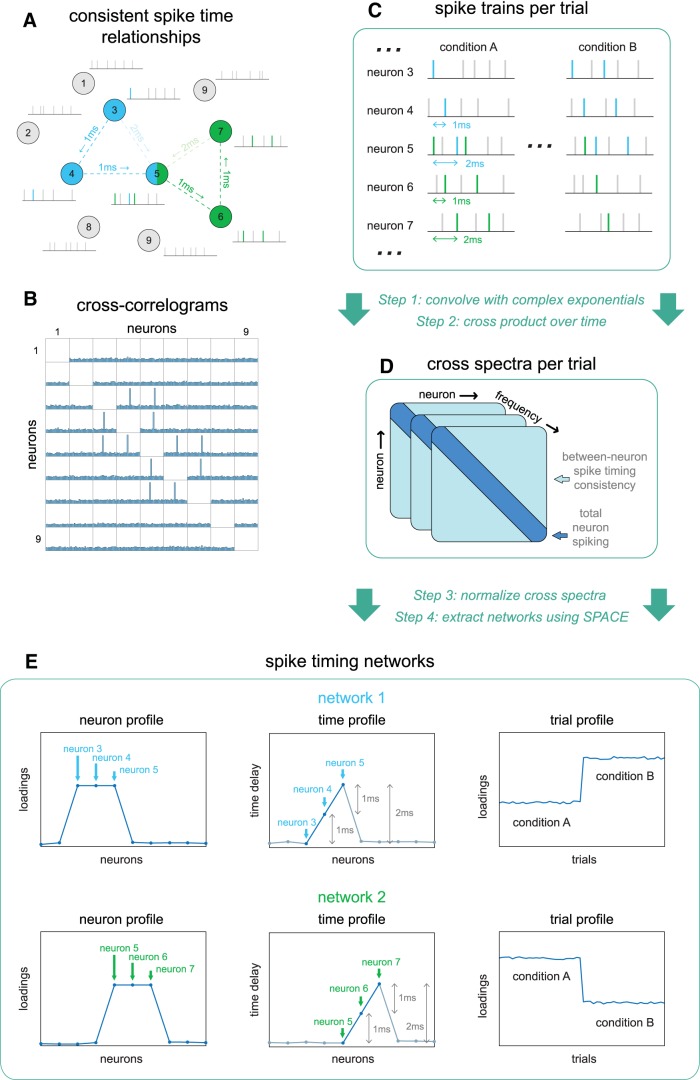
Schematic of extracting spike timing networks. Neuronal spiking time series can contain consistent spike timing between neurons, forming spike sequences. ***A***, Schematic of two spike timing networks, with their neurons (circles) and sequence spikes (vertical lines) colored blue and green. The dashed lines reflect the between-neuron consistent spike time relationships resulting from the spike sequences. The blue network’s sequence goes from neuron 3–4–5 (dark dashed lines), with 1 ms time delays, resulting in a 2 ms delay from 3 to 5 (light dashed line). The green network’s sequence is the same but from neuron 5–6 to 7. ***B***, The spike time consistencies in ***A*** can also be visualized as cross-correlograms between all neuron pairs, at lags ranging from -10 to 10 ms with 1 ms bins. ***C***, The networks in ***A***, ***B*** but shown as spike trains per trial of two experimental conditions. The blue network trials’ have one sequence in condition A and two sequences in condition B, vice versa for the green network. To extract these two networks, we arrange spike trains of all neurons in a neuron-by-time binary matrix. These spike trains are then convolved with complex exponentials (or wavelets) of equal length at different frequencies, resulting in a complex-valued neuron-by-time matrix per frequency per trial. ***D***, The cross products are then computed along the time dimension, resulting in a neuron-by-neuron cross-product matrix per frequency per trial: the cross spectrum. The between-neuron phase differences of the cross spectra over frequencies, reflect the consistent between-neuron spike time delays. ***E***, Using a recent technique denoted as SPACE, the structure in the cross spectra over frequencies can be extracted, and described as separate spike timing networks. The blue and green networks are each described by a neuron profile, describing network membership by a single weight per neuron, a time profile, describing the spike sequence by a time coefficient per neuron, and a trial profile, having a single weight per trial, indicating how strongly the network was present. For details, see Materials and Methods, sections Extracting spiking timing networks from neuronal spike recordings, SPACE describes time consistency-induced phase coupling in cross spectra, and Obtaining cross spectra that are optimal for extracting spike timing networks.

We present a novel approach for revealing spike timing networks that does not suffer from the above problems. The key features of our approach, are that (1) it can find networks regardless of the complexity of their spiking sequences, and that (2) it describes these sequences with sub-millisecond precision by a time coefficient per neuron (per sequence, see Fig. 1). This is achieved by applying a method developed for electrophysiological recordings (intended for revealing phase-coupled oscillatory networks, such as traveling waves; van der Meij et al., 2015), on the spectral covariance (or cross spectra) obtained from a spectral analysis of discrete neuronal spiking time series. In these cross spectra, consistent between-neuron spike time delays are described by linearly increasing phase differences over frequencies. The method finds networks and their spike sequences by their unique patterns of between-neuron phase differences over frequencies and trials (epochs). In the following, we first show that our approach is capable of recovering simulated spike timing networks and their sequences under various noise conditions, and then provide a proof-of-concept by showing networks extracted from rat hippocampus and medial prefrontal cortex (Fujisawa et al., 2008, 2015), which reflected peak cross-correlogram time delays with high accuracy. Together, this demonstrates that our approach is a robust method for revealing and characterizing spike timing networks in neuronal recordings.

## Materials and Methods

### Extracting spiking timing networks from neuronal spike recordings

Spike timing consistency between neurons is thought to be a key feature of neuronal spike recordings. In the following, we describe how a novel application of a recent technique, SPACE (van der Meij et al., 2015, 2016), can be used to find sequences of consistent time-shifted spikes between neurons, denoted in the following as spike timing networks, in large scale neuronal spike recordings, without a priori information about the involved neurons and their timing. Below, we illustrate the procedure for extracting spike timing networks (with details in respective sections) and how to interpret their characterization.

We start with any kind of multielectrode neuronal recording over time t of which neurons J and their spikes have been identified (using, e.g., Rossant et al., 2016). Suppose our recording contains two groups of three neurons that have consistent between-neuron spike timing (i.e., spike timing networks; Fig. 1*A*, blue/green, *B*), embedded in other spiking activity. The blue network has a spiking sequence of neuron 3-4-5 with a timeline of 0–1–2 ms, leading to consistent spike time delays of 1 ms for pairs 3-4 and 4-5, and a 2 ms delay for pair 3-5. The green network has the same pattern but for neurons 5, 6, and 7. If cross-correlograms were computed from the neuronal spike recordings (Fig. 1*B*), they would have peaks at 1 ms for neuron pairs 3-4, 4-5, 5-6, and 6-7, at -1 ms for pairs 4-3, 5-4, 6-5, and 7-6, at 2 ms for pairs 3-5 and 5-7, and at -2 ms for pairs 5-3 and 7-5. How often the sequences of the blue and green networks occur depends on two experimental conditions. The blue networks occur once per trial of condition A and twice for those of B (Fig. 1*C*), the green network vice versa.

To extract a parsimonious description of the above networks, we arrange the detected spikes in neuron-by-time (J×t) binary matrices $S_{l}$ (1 = spike, 0 = no spike; Fig. 1*C*), per trial l of the experiment (or any other meaningful temporal segmentation; throughout the text, J refers to neurons, K to frequencies, L to trials). Then, we obtain “cross spectra” from these trial-specific matrices. To achieve this, we first convolve the matrices $S_{l}$ with complex exponentials (Fig. 1, step 1) at multiple frequencies k, resulting in frequency-specific and trial-specific complex-valued neuron-by-time (J×t) matrices $Z_{kl}$. Subsequently, we compute the cross products $Z_{kl}Z_{kl}^{*}$ of these matrices over time (Fig. 1, step 2; * = complex conjugate transpose), resulting in complex-valued frequency-specific and trial-specific neuron-by-neuron (J×J) matrices $X_{kl}$: the cross spectra (Fig. 1*D*). The choice of complex exponentials determines key aspects of the spike timing networks and their extraction (for details, see below, Obtaining cross spectra that are optimal for extracting spike timing networks). Having obtained the cross spectra, we should apply a neuron-wise and/or trial-wise normalization (Fig. 1, step 3; see below, Normalizations of the cross spectra). After normalization, we then extract spike timing networks using SPACE (Fig. 1*E*, step 4; which involves estimating the number of networks to extract, see below, SPACE describes time consistency-induced phase coupling in cross spectra).

Spike timing networks, when defined by their discrete spiking sequences characterized as time-shifted copies of spikes between neurons, can be extracted from the cross spectra, because the phases of the off-diagonal elements of the cross spectra contain the consistent time delays between time-shifted copies of spikes. The crucial principle here, is that the time difference between two binary spikes in the time domain translates to phase differences in the frequency domain, linearly increasing with frequency. That is, a 1 ms time delay equals 1/20th of a cycle at 50 Hz, 1/10th at 100 Hz, 1/5th at 200 Hz, etc. (for types of spiking interactions other than time-shifted copies of spikes, see Lindemann et al., 2001). The extraction technique uses this property to find the time delays between time-shifted copies of neuronal spikes that explains the most variance in the cross spectra.

SPACE describes the cross spectra by multiple networks, each network consisting of three parameter vectors (Fig. 1*E*): the neuron profile (1×J), the time profile (1×J), and the trial profile (1×L). The neuron profile describes how strongly each neuron is part of the network, by a single number per neuron. The neuron profile of the blue network has high values for neurons 3, 4, and 5 and low values for all other neurons. That is, only neurons 3-5 are part of this network. Due to their similar weighting, neurons 3-5 likely have similar firing rates, and similar number of spike sequence’ spikes. If another neuron would have half the weighting, it will likely have either twice as many total spikes, or only fires in half of the network’s spike sequences (e.g., a sequence of neuron 3-4-5 in half of the trials and 3-4 in the rest). Note, it is possible for the neuron profile to have non-zero loadings for only one neuron (for a discussion, see below, Normalizations of the cross spectra). The time profile describes the spike sequence of the network, by a single time coefficient per neuron. Because the neuron profile of the blue network only strongly involves neurons 3-5, only these coefficients of the time profile are meaningful. The time profile of neurons 3-5 directly reflects the timeline of the spike sequence, 0–1–2 ms. A crucial observation here, is that all the temporal relationships at the level of neuron pairs are described at the level of individual neurons by the network’s neuron profile (relationship strength) and time profile (spike sequence timing). The above is the same for the green network. Importantly, if the blue and green spike sequences occur at random intervals between them, the blue and green networks can be separated, as there are no consistent relationships between neurons 3 and 4 and neurons 6 and 7. If the blue and green spike sequences occurred at consistent intervals, they would both be captured by one network. This is not surprising, as the above means there is, in fact, only one spike sequence. In the schematic of Figure 1, there is also a difference in how often a sequence of each network occurs in each condition, which can be reflected by the trial profiles. Here, the weights for trials of the blue network reflect the ratio of spike sequences in each trial of the two conditions, i.e., a trial loading that is twice as large for B as it is for A. Importantly, the trial profile can provide a convenient way to investigate differences in spike time consistency at the level of networks, instead of the level of neuron pairs. For example, the difference between two conditions can be investigated by comparing the means of the condition-specific trial profile weights, or variations of trial profile weights can be related to other variables (e.g., reaction times, parametric stimulus manipulations, etc.). Although in principle possible, the trial profile can be noise sensitive (see Results, Discussion). Additionally, the absence of an extracted network is not evidence of a network’s absence (in the recording), various reasons can prohibit a network to not be found (e.g., noise; see Results, Discussion). Finally, see below, SPACE describes time consistency-induced phase coupling in cross spectra, on how to compare values between and within the above profiles.

When interpreting the network profiles, it is crucial to keep in mind that they are estimated to maximally explain the cross spectra (see below, SPACE describes time consistency-induced phase coupling in cross spectra). As such, anything that affects the phase coupling patterns in the cross spectra, affects the profiles accordingly. For example, in case a neuron spikes in a spike sequence of a network in some trials, but not in others, then the phase coupling strength between this neuron and the others of the network will be weaker (or zero) in the cross spectra for the latter trials compared to the former. Consequently, the neuron profile of this network will have a lower weight for this neuron than for the others, and the trial profile will have lower weights for those trials in which it did not spike.

### SPACE describes time consistency-induced phase coupling in cross spectra

SPACE is a decomposition technique that describes the structure of phase coupling in cross spectra by time delays between neurons (or electrodes/sensors/sites). The technique was developed for finding oscillatory phase coupling structure (e.g., traveling waves) in electrophysiological recordings (van der Meij et al., 2015, 2016), a type of data of which the frequency content itself is of primary interest. This contrasts with the application we present here, for which this is not the case. The frequencies of the used spectral transform are artificial and are chosen only such that they provide an accurate description, via the networks SPACE extracts, of the temporal structure in discrete neural spike timing time series (see below, Obtaining cross spectra that are optimal for extracting spike timing networks). Apart from the manner of constructing the cross spectra, the usage of the method in the current approach is identical to that in the original publication (referred to as SPACE-time therein). The algorithm behind the method is extensively treated in its original publication (van der Meij et al., 2015; but for an alternate presentation, see van der Meij et al., 2016), and only elements essential to its current use will be mentioned here. Briefly, the technique consists of an alternating least squares (ALS) algorithm to find the least squares estimates of its decomposition model. The element-wise formulation of this model for the cross spectra can be expressed as:

$$X_{j_{1}j_{2}kl}=\overset{F}{\underset{f=1}{\sum }}(a_{j_{1}f}\,\;\cdot \;a_{j_{2}f})\;\cdot \;exp(i2\pi \varphi_{k}\left(\sigma_{j_{1}f}-\sigma_{j_{2}f}\right))\;\cdot \;b_{kf}^{2}\,\;\cdot \;c_{lf}^{2}+\varepsilon_{j_{1}j_{2}kl}$$

The complex-valued cross spectrum ($X_{j_{1}j_{2}kl}$) of neuron pair $j_{1}$ and $j_{2}$ (indexed over all neurons) at frequency k and trial l is described as the product of network parameters, summed over networks F, plus an error term $\varepsilon_{j_{1}j_{2}kl}$. The phase of the network-specific product is given by the difference in the time profiles ($\sigma_{j_{1}f}-\sigma_{j_{2}f}$) of neuron $j_{1}$ and $j_{2}$ multiplied by the frequency $\varphi_{k}$ in Hz, multiplied by 2π. This phase is then weighted by the product of the two neurons’ neuron profile $a_{j_{1}f}\cdot a_{j_{2}f}$, the (squared) frequency profile $b^{2}$ at frequency k, and the (squared) trial profile $c^{2}$ at trial l. As is observed here, the technique also produces a frequency profile per network, describing how important each frequency is for a network. For the purpose of spike timing networks, we will ignore this, as it does not provide additional information. It is, however, an essential element of its original application on electrophysiological recordings, describing frequency band-specific phase-coupled oscillatory networks, such as traveling waves.

Compared to the reference publications, the above equation squares the trial and frequency profile. The reason for this, is that spike timing networks are more conveniently thought of, analyzed at, and simulated in, the description level of cross spectra. This is not the case for the original target of the technique, phase-coupled oscillatory networks, which are more conveniently thought of as time-varying oscillations over electrodes described by Fourier coefficients (of which the cross products over time produce the cross spectra). Due to this, the technique provides trial and frequency profiles that are not squared, and squaring becomes a necessary step before investigating the extracted networks.

The extracted networks are unique up to trivial indeterminacies without requiring constraints such as orthogonality or statistical independence. Uniqueness is discussed in more detail in the reference publications (van der Meij et al., 2015, 2016). The indeterminacies are easily resolved by normalizations. Here, we briefly highlight those normalizations that pertain to the current application of the technique. The neuron and trial profiles, per network, have undetermined multiplicative scaling, and are normalized to have a vector L2 norm of 1. The consequence is that the absolute values of neurons and trials only have meaning with respect to the other neurons and trials of the same network. Crucially, the ratios between neurons and trials are unaffected by this normalization and can be compared freely across networks. Additionally, their sign is also undetermined, and restricted to have a positive average per network (neuron profile) or to be fully positive (trial profile). The indeterminacy of the time profile is more complicated. Because the time profile describes circular phases over multiple frequencies, the time profile is circular as well. In short, we normalize it such that the strongest neuron (of the neuron profile) has a time profile value of 0 s. Due to the above normalizations, a network-specific multiplicative scaling parameter is also extracted, but it does not play a role in the interpretation of the individual network parameters.

Two practical points need to be made for using the technique to extract networks. The first is that its algorithm is initialized from random starting values. to avoid unfortunate starting values that lead to a local minimum of its least squares loss function, the algorithm needs to be initialized multiple times. When identical networks are found in those initializations with the highest explained variance, it can be assumed that the global minimum is reached. How many initializations are required to achieve this depends on the particular dataset. In our experience, it is extremely rare to find a different “best” solution to the loss function when increasing the number of random initializations beyond 50. The second practical point is that, like related decomposition techniques, the number of networks to extract needs to be determined. One approach is to estimate the number of reliable networks. For this, we extract *N* networks from the full recording, and also from two splits of the recording, the first containing the odd numbered spikes of each neuron, the second the even numbered spikes. If the networks from the full recording reasonably match those extracted from of both splits, *N* is increased, and the process is repeated until they no longer match. The networks extracted from the full data are kept, and those of the splits discarded. Other scenarios are also feasible, such as an odd-even trial split, or a k-split approach, in which networks are extracted from k subsets of the recording and compared to those extracted from the full recording. The number of splits, and the manner of splitting, will determine the sensitivity of the approach. It is useful here to make a technical statement regarding the splitting of individual networks into two or more smaller networks, which would complicate the above. to avoid such splitting, network interaction terms in the decomposition model are forced to be zero (interaction terms are not visible in the element-wise model above; for optimization details, see van der Meij et al., 2015, 2016). A practical consequence is that spike timing networks that are nearly perfectly correlated (share spike sequence timing) will likely be extracted as a single combined network (for simulations investigating network correlation, see van der Meij et al., 2016). Finally, regarding the maximum number of networks that can be uniquely extracted, although nontrivial to estimate (Comon et al., 2009), it likely is greater than the number of neurons. Importantly, any kind of reliability procedure such as the above will prevent exceeding any maximum, as nonunique networks will, by definition, not be reliable over splits. In our experience, the number of reliable networks is often much lower than the number of recorded neurons.

To determine whether two networks are similar, such as in the above split-reliability approach, a coefficient can be computed for the three parameters of the networks. For the neuron and the trial profiles, this is simply the inner product between the L2-normalized profiles of two networks, and ranges from 0 to 1 (identical profiles). For the time profile, a coefficient is the following:

$$time\,\;profile\,\;similarity:\left|\langle A^{1}\,\;\cdot \;exp\left(i2\pi \gamma \sigma^{1}\right),A^{2}\,\;\cdot \;\overset{¯}{exp\left(i2\pi \gamma \sigma^{2}\right)}\rangle \right|$$

Time profile similarity is computed as the absolute value || of the inner-product ⟨,⟩ over neurons J of the time profiles σ of two networks (superscript 1 and 2, ¯ denotes complex conjugate), weighted by the normalized neuron profiles A of each network (· denotes the element-wise product). Here, γ stands for the greatest common divisor of the frequencies used to extract the networks, in Hz, which determines the “cycle length” of the circular time profile (van der Meij et al., 2015). This similarity coefficient also ranges from 0 to 1 (identical profiles). Allowing for some differences in the profiles due to noise, we considered networks similar enough when coefficients for the neuron, time, and trial, profiles are all equal to, or greater than, 0.7.

#### Software and code accessibility

The technique is freely available in a public GitHub repository termed nwaydecomp (www.github.com/roemervandermeij/nwaydecomp), together with tutorials on its use. The toolbox also contains software to deal with the practical points above. The code is also available as Extended Data Figure 1.

### Obtaining cross spectra that are optimal for extracting spike timing networks

To be able to extract spike timing networks we compute cross spectra from binary spike trains, by convolving the spike trains with complex exponentials (“wavelets”) and computing their cross products over time (Fig. 1, step 1–2). Doing so transforms the time delays between spikes of different neurons, into phase differences at multiple frequencies. The length of the complex exponentials, and their frequency, determines how sensitive the cross spectra are to consistent versus nonconsistent time delays, and is described in the following. Here, it is important to keep in mind that the cross spectrum between two neurons, is exactly the complex-valued sum, of the phase differences between spikes of neuron 1 and spikes of neuron 2 that are overlapping after the convolution, weighted by their amount of temporal overlap. Due to the latter, long time delays necessarily have lower weighting in the cross spectra than short time delays.

Obtaining the cross spectra can be expressed as follows:

$$X_{j_{1}j_{2}kl}=Z_{j_{1}kl}Z_{j_{2}kl}^{*}$$

$$Z_{jkl}=S_{jl}⊗exp\left(iT2\pi \varphi_{k}\right)$$

That is, the cross spectrum between neuron neurons $j_{1}$ and $j_{2}$ at frequency k and trial l is obtained as the cross product, over time, of vectors $Z_{j_{1}kl}Z_{j_{2}kl}^{*}$ (* = complex conjugate transpose). The vector $Z_{jkl}$ for neuron j is obtained by the convolution ⊗ of the binary spike train vector $S_{jl}$ with a complex exponential (untapered wavelet, i is the imaginary unit) at frequency $\varphi_{k}$. Here, T is a vector of time points from −t/2 to t/2, with t being the time domain length of the complex exponential, and both $S_{jl}$ and T are sampled at the maximum achievable sampling rate. Note, the edges of $Z_{jkl}$, for which the complex exponential was not fully immersed in $S_{jl}$, are kept.

The time domain length t of the complex exponentials determines which between-spike time delays can contribute to the cross spectra, and it should be chosen based on the expected range of time delays. Here, we aim to be sensitive to time delays of 0±10 ms, a range that captures commonly occurring consistent spike timing (Nelson et al., 1992; Fujisawa et al., 2008; Sakurai et al., 2013). The optimal time domain length for this range is a trade-off. The longer the length, the lower the sensitivity will be to the expected time delays, as the cross spectra will reflect a sum of more spike pairs. The shorter the length, the bigger the ratio between the weighting (samples overlap) of the shortest and the longest expected time delay, and thus the stronger the bias in sensitivity toward the former. As a compromise between the two, we choose a time domain length of twice of the maximum time delay we wish to be sensitive for 20 ms. This results in an overlap of 50% of the complex exponentials’ samples for spikes at a delay of ±10 ms, having an acceptable sensitivity bias ratio of 0 ms (shortest; 100% overlap) to ±10 ms of 2:1 (compared to 400:1 for 0±20 ms at a sampling rate of 20 kHz). When there is no a priori expectation regarding the length of the time delays, the time domain length t can be based on, e.g., an investigating of the peak of the between-neuron cross correlograms. In general, it is preferable to choose a length t that is too long rather than too short, as the sensitivity cost due to additional spike pairs in the cross spectrum is much less than that of (1) a more skewed sensitivity bias ratio and (2) the experimental cost of spike time consistency at longer delays being invisible. to further reduce the bias of short time delays to long time delays, the complex exponentials should have constant magnitude, and not be tapered using a particular windowing function (such as a Hanning window).

The frequencies φ of the complex exponentials greatly determine the sensitivity of the cross spectra to nonconsistent time delays. to be maximally sensitive to consistent time delays, the contribution to the cross spectrum of all other time delays should be as small as possible. In the terms of phase differences in the cross spectra, this is achieved when the average of the complex-valued phase differences of the nonconsistent time delays approaches a magnitude of 0. This is the case for any frequency whose cycle length is an integer multiple of the time domain length chosen above (for 20 ms, 50, 100, 150 Hz, etc.), under the assumption that nonconsistent spike pairs are equally likely at any time delay. To arrive here, it is crucial to appreciate the fact that phase differences for large time delays are weighted lower than those for small time delays. For phase differences originating from time delays between 0 ms and the time domain length (20 ms above) to have an average magnitude of 0, the weighting coefficients for phase differences between π/2 and −π/2, the left side/quadrant 2 and 3 of the unit circle, need to have the same sum as those for −π/2 to π/2, the right side/quadrant 1 and 4 of the unit circle. Crucially, for the frequency whose cycle length equals the maximum time delay, the 25% smallest time delays fall in quadrant 1, the middle 50% of time delays fall in quadrant 2 and 3, and the 25% largest time delays fall in quadrant 4. Equally crucial, the weighting is a linear function of the time delays. As for any linear function, the sum of the first 25% and the last 25% of a subset of its values is equal to the sum of its middle 50%, the frequency with a cycle length equaling the largest time delay will have phase differences from nonconsistent time delays that approach an average magnitude of 0. This also holds for any integer multiple *N* of this frequency, as the above will be the case for *N* equal splits of the time delay range. Note that to obtain an average magnitude of 0, it is also required that the sum of weighting coefficients for quadrant 1 and 2 (top of unit circle) is equal to that of quadrant 3 and 4 (bottom of unit circle). This symmetry is easily achieved however, as the weighting coefficients for -20 to 0 ms progress along the unit circle in opposite direction than those for +20 to 0 ms. As a last note, the frequencies of the complex exponentials also determine the robustness to jitter around a consistent time delay. The lower the frequency, the closer the phases of jittered but consistent time delays, the higher their average magnitude, and thus, the more robust to jitter.

Concluding, we compute cross spectra by convolving spike trains with complex exponentials of 20 ms in length, constant magnitude, and at 20 frequencies from 50 to 1000 Hz in steps of 50 Hz. When investigating longer time-scale neuronal dynamics, an analogous set would be, e.g., 1 s length, at 1–20 Hz in steps of 1 Hz. The number of frequencies to use is somewhat arbitrary. Initial simulations showed no noticeable difference beyond 20 frequencies (and 1000 Hz is already very sensitive to jitter), and simulated networks were reliably recovered from simulations as little as five frequencies.

### Normalizations of the cross spectra

The neuron profiles of spike timing networks describe the off-diagonal elements of each cross spectrum, reflecting between-neuron spike pairs, and the diagonal elements, i.e., power, reflecting the total number of spikes of neurons. In realistic data, the firing rates of neurons can differ greatly, resulting in large differences in power. Because the power of each cross spectrum is typically much larger than its off-diagonal elements, this can lead to spike timing networks whose neuron profiles are driven more by firing rates of individual neurons, rather than consistent spike timing between neurons. An extreme example is a neuron profile with a non-zero weighting for only a single neuron. Such a “network” only describes the diagonal element (i.e., firing rate) of the cross spectra of the respective neuron, and should be considered as an artefactual network. To increase sensitivity to consistent spike timing, to avoid the above, power differences between neurons can be normalized (Fig. 1, step 3). Normalizing power such that it is equal to an *N*th root, summed over frequencies and trials, is one such normalization:

$$neuron-wise\;normalization:\frac{X_{kl}=W^{\frac{1}{2}}X_{kl}W^{\frac{1}{2}}}{W=\frac{\sum_{K}\sum_{L}{\left[X_{kl}^{diag}\right]}^{\frac{1}{N}}}{\sum_{K}\sum_{L}\left[X_{kl}^{diag}\right]}}$$

Here, $X^{diag}$ is a diagonal matrix containing only the diagonal elements of X. By increasing *N*, the power differences between neurons decrease. Ideally, the power of every neuron becomes equal, i.e., the cross spectra become coherency spectra, as this will have the highest sensitivity to spike time consistency. However, this can have the unintended consequence of interfering with the split reliability procedure for estimating how many networks to extract. Briefly, when extracting fewer than the total number of networks, which networks are extracted strongly depends on their explained variance; those with the most, tend to be extracted first (as the networks are found by a randomly initialized least squares algorithm). When the differences in explained variance between networks decrease, the order in which they are extracted becomes more variable, which can prematurely stop the split reliability procedure. Increasing neuron-wise normalization strength can result in decreasing differences in explained variance. As such, while neuron-wise normalization increases the usefulness of the networks, it can also result in finding less split-reliable networks. Practically, an optimal normalization strength can be found as follows. First, a split reliability procedure is run with normalization strength *N* = 1 (no normalization). If this results in (1) split-reliable networks and (2) networks that are unlikely to reflect spike time consistency (neuron profiles that have strong weighting for only one neuron), the normalization strength *N* is doubled and the split reliability procedure repeated. This is repeated until sufficient reliable networks are found that reflect spike consistency. A convenient quantification of when networks are unlikely to reflect spike time consistency is to compute the ratio of the strongest and second strongest weights of the neuron profile; the higher this ratio, the more likely the second strongest weight reflects noise and that the network does not reflect spike time consistency. Then, in the above procedure, a cutoff ratio of 5-to-1 can be used as a conservative criterion (see also Results, Spike timing networks extracted from real recordings reflect between-neuron spike timing relationships). Crucially, the above only affects the probability of uncovering those spike timing networks that already exist in the recording, the phase coupling structure in the cross spectra induced by the networks remains unaffected.

The firing rate of neurons can also differ greatly between trials. Because the trial profile reflects variations in the cross spectra over trials in its weights, it reflects both the trial-specific firing rate of the involved neurons as well as their trial-specific amount of spike timing consistency. Similar to the above, normalizing the cross spectra over trials can reduce the impact of firing rate on the trial profile. Normalizing cross spectral power such that it is equal across trials is one such normalization:

$$trial-wise\;normalization:\frac{X_{kl}=W_{kl}^{\frac{1}{2}}X_{kl}W_{kl}^{\frac{1}{2}}}{W_{kl}=\frac{1}{X_{kl}^{diag}}\sum_{L}X_{kl}^{diag}}$$

As above, $X^{diag}$ is a diagonal matrix containing only the diagonal elements of X. Importantly, in the common case of a neuron not spiking in a particular trial, its elements of the cross spectra (X) will be zero, leading to division-by-zero errors during the above normalization. This is avoided by adding random noise of trivial strength (close to the used numerical precision) to the respective elements of the cross spectra before normalization. Trial-wise normalization is achieved by first normalizing frequency- and trial-specific cross spectral power to 1 (by division by itself), and then multiplying it with the frequency-specific summed power over trials $(\underset{L}{\sum }X_{kl}^{diag};\;computed\,\;\;before\,\;normalization)$. Trial-wise normalization is independent of the above neuron-wise normalization, both normalizations can be applied jointly. The normalization above is extreme, as it removes all cross spectral power variability over trials. However, because normalization occurs via a diagonal matrix (as is the case with neuron-wise normalization), the off-diagonal elements of the cross spectra only undergo a scaling proportional to their diagonal elements; their magnitudes still reflect the (relative) amount of spike timing consistency between their involved neurons. As such, the remaining trial-by-trial variations in cross spectral magnitudes maximally reflects trial-by-trial variations in the amount of spike timing consistency. Similar to the neuron-wise normalization, the trial-wise normalization can make it harder to uncover networks that are present in the recording. This can be dealt with more conveniently however. First, networks are extracted using a split reliability procedure as discussed above, without trial-normalizing cross spectra. Once reliable networks are obtained, the trial profiles are re-estimated in one final decomposition using cross spectra that are additionally trial normalized, in which the neuron and time profiles are kept constant. Although the above two-step approach is advised, in the case of our simulations the differences were negligible, and for simplicity the results that are presented were extracted from trial-wise normalized cross spectra in one step.

### Simulating and extracting noisy spike timing networks

To investigate the effects of various kinds of noise on spike timing network extraction, we simulated spike recordings of 15 neurons at 100 trials of 1 s containing four spike timing networks. Network spiking sequences had a fixed temporal structure that was repeated between zero and three times (predetermined) per trial (1.2 Hz average spike sequence rate for each network). Within each trial, each spike sequence could occur anywhere with uniform probability, with a 25 ms offset from trial boundaries. On trials where spike sequences of multiple networks were present their order was randomized, and with a minimum of 25 ms between sequences. Three kinds of noise were simulated. First, all neurons of a single simulation had a noise spiking rate of 0, 5, 10, 20, or 100 Hz, as Poisson spiking superimposed on the network spike sequences. Second, each spike of each spike sequence could have an individual random jitter (uniformly distributed) at a maximum of 0, ±0.25, ±0.5, ±1, or ±2 ms. Third, each spike in each spike sequence occurrence had an individual deletion probability of 0%, 10%, 20%, 40%, 80%, resulting in partial spike sequences. Cross spectra of each simulation run were obtained as described above, using a time-window length of 20 ms and frequencies from 50 to 1000 Hz in steps of 50 Hz. The four networks were extracted using 10 random initializations of the extraction algorithm. Note that the purpose of these simulations is to show how well spike sequences can be extracted under noisy conditions, and not how such a pattern can be generated physiologically, nor whether such a pattern is physiologically meaningful. As such, we simulated data from the perspective of spikes, instead of model neurons generating spikes, which also provides a convenient ground truth for calculating recovery.

### Quantifying recovery of simulated spike timing networks

To quantify the recovery of the extracted neuron profile, time profile, and the trial profile, they were compared to their simulated equivalents. The simulated neuron profiles were constructed as a binary 1×J vector per network, its values indicating network membership of each neuron. Similarly, simulated trial profiles were constructed as 1×L vector, its values reflecting the number of sequence repeats (linear modulation of network activity over trials). Finally, simulated time profiles were constructed as a 1×J vector, its values describing the temporal sequence of spikes in seconds (nonmember neurons set arbitrarily to 0). For display purposes these simulated parameters were normalized in the same manner as the extracted network parameters. to compute recovery, extracted networks were paired to the simulated networks using the similarity coefficients described above, by first determining the most similar pair, then the next most similar in the remainder, etc. Recovery of neuron and trial profiles was determined using a Pearson correlation coefficient. Time profile recovery was judged by the following coefficient:

$$time\,\;profile\,\;recovery:\frac{\left|\underset{J}{\sum }exp(i2\pi \gamma \sigma^{e})\;\cdot \;\overset{¯}{\;exp\left(i2\pi \gamma \sigma^{s}\right)}\,\;\cdot \;A^{s}\right|}{\underset{J}{\sum }A^{s}}$$

Time profile recovery is computed as the absolute value || of the weighted sum over neurons J of the complex-valued difference of the circular time profiles σ of the extracted and simulated networks (superscript *e* and *s*, respectively; ¯ denotes complex conjugate), weighted by the simulated neuron profile A (· denotes the element-wise product). Similar to the similarity coefficient described above, γ is the greatest common divisor of the frequencies used for extraction (i.e., 50 Hz), and is used to deal with the circularity of the time profile. This coefficient ranges from 0 to 1 (perfect recovery).

### Extracting spike timing networks from recordings of rat medial prefrontal cortex and hippocampus

As a proof of principle, we extracted spike timing networks from real spiking recordings, obtained from a dataset publicly available at http://crcns.org/ (Fujisawa et al., 2008, 2015). This dataset contains identified neurons and theirs spikes from recordings obtained from rat medial prefrontal cortex and area CA1 of the hippocampus, while the rat performed an odor-based delayed matching-to-sample task, requiring it to run through either the left or right arm of a maze to obtain its reward. Animal recording protocols were approved by the Institutional Animal Care and Use Committee of Rutgers University, Newark, NJ. The recording used (rat GG.069) came from eight and four electrode shanks (200 µm shank separation) in medial prefrontal cortex and CA1, respectively, each shank containing eight contacts (20 µm contact separation; 160 µm^2^ contact surface). The recording was sampled at 20 kHz, and offline spike sorting was performed (after band-passing between 0.5 and 5 kHz) using KlustaKwik (for spike sorting details, see Fujisawa et al., 2008, 2015). A total of 63 neurons were identified on nine shanks. The dataset contained 20 left and 20 right trials, having an average duration of 8.04 s (SD = 1.73 s). Only neurons with average spiking rates of 1 Hz and above were selected. To extract networks, we first obtained cross spectra as described above (Obtaining cross spectra that are optimal for extracting spike timing networks), using a time window of 20 ms and frequencies from 50 to 1000 Hz in steps of 50 Hz, dividing each cross spectra by its trials’ duration. Subsequently, cross spectra were neuron-wise normalized as described above. A normalization such that cross spectral power was equal to its 32nd root normalization was chosen as an optimal normalization, because 64th root normalization resulted in no split reliable networks (likely due to varying network order mentioned above (Normalizations of the cross spectra), and 16th root normalization resulted in many networks mostly consisting of single neurons (i.e., the bias toward power differences between neurons was too strong to overcome). The number of networks to extract was determined using odd-even spike split reliability procedure described above (SPACE describes time consistency-induced phase coupling in cross spectra). With a similarity coefficient cutoff of 0.7, 50 random initializations were used at each step. This resulted in four networks being extracted. Continuous cross-correlograms were obtained at time lags of ±20 ms at 0.05 ms steps by summation of the (lagged) binary spike trains after they were convolved with a Gaussian with full-width at half-maximum of 0.5 ms (maximum = 1).

## Results

Spike timing networks consist of multiple neurons that have consistent time delays between their spikes, forming a spike sequence. Here, we validate a novel approach for finding and characterizing these networks in neuronal spike recordings. First, we evaluate its robustness to various noise conditions. We show how the recovery of simulated spike timing networks is affected by spike jitter in the network spike sequences and variability of neuron participation in the network, under increasing spiking noise of all simulated neurons. Then, we show how variable firing rates of neurons affects recovery, and what actions can be taken to reduce negative effects. Finally, we provide a proof-of-principle by showing networks extracted from rat hippocampus and medial prefrontal cortex (Fujisawa et al., 2008, 2015), and compare the extracted spike timing relations to cross-correlograms of the involved neurons.

### Simulated spike recordings from spike timing networks

**Figure 2.**
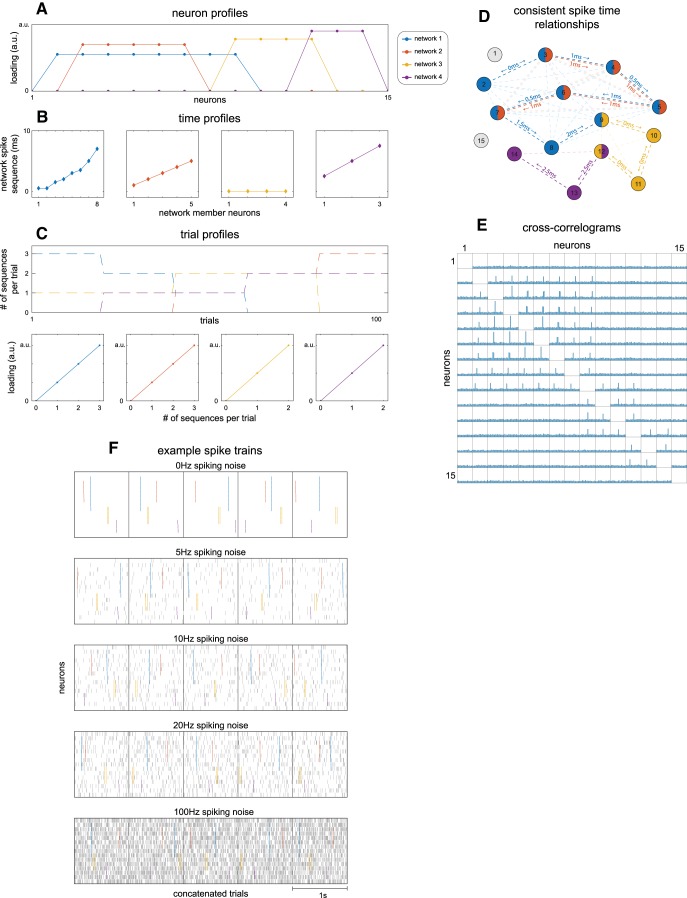
Simulated spike timing networks. To investigate the robustness of network extraction to various kinds of noise, we simulated spike recordings from 15 neurons containing four spike timing networks across 100 trials. ***A–C***, Description of simulated networks in same format as extracted networks. ***A***, The neuron profile of each network describes nonmember neurons by 0 s and member neurons by 1 s. Because absolute magnitudes of the neuron and trial profiles of networks are not meaningful, they are L2 normalized by convention (leading to the visible arbitrary between-network amplitude differences); the between-neuron/trial ratios are meaningful. ***B***, The spiking sequence of each network, shown as their time profiles (only member neurons are shown). ***C***, The trial profile. Each network spiking sequence was repeated zero to three times in each trial, shown per trial in the first row. The second row of ***C*** shows an alternative visualization of the trial profile, which is convenient for visualizing recovery (Figs. 3–6). Here, normalized trial profile weights (*y*-axis) are shown as their mean (SD), per simulated number of spike sequence repeats (*x*-axis). ***D***, Schematic of all consistent spike timing relationships resulting from the simulated spike sequences. Each circle is a neuron, each dashed line reflects a spike timing relationship. For visibility, the first-order relationships are dark colored, all others are light colored. Numbers indicate the first-order within-sequence spike time delays. ***E***, Cross-correlograms computed for all neuron pairs from a simulation run with 20 Hz spiking noise, at lags ranging from -10 to 10 ms with 1 ms bins. ***F***, Raster plots of example spike trains as a function of spiking noise levels used in the simulations. Each vertical dash is a single spike. Each row consists of five concatenated trials, separated by a vertical line. Network spiking sequences are shown by their color as in ***A–D***. See Materials and Methods, Simulating and extracting noisy spike timing networks.

To investigate the robustness of spike-timing network extraction to various kinds of noise, we simulated spike recordings of 15 neurons over 100 trials of 1 s containing four networks (for simulation details, see Materials and Methods, Simulating and extracting noisy spike timing networks). A network was defined as a group of neurons that spike in sequence, with between-spike time delays ranging from 0 (synchronous) to 2.5 ms. The spike sequence timelines were 0–0–1–1.5–2.5–3–4.5–6.5 ms for network 1, 0–1–2–3–4 ms for network 2, 0–0–0–0 ms for network 3 (all synchronous), and 0–2.5–7.5 ms for network 4. Each network’s spike sequence was repeated zero to three times in groups of trials to simulate linear modulations of network activity across the task. Some of the networks had neurons that were involved in other networks’ spike sequences: all of the neurons of the spike sequence of network 2 were also part of network 1, and one neuron was shared between network 1 and 3, and network 3 and 4. In Figure 2*A–C* we show the networks’ neuron profiles (Fig. 2*A*), time profiles (Fig. 2*B*), and trial profiles (Fig. 2*C*). The simulated recordings of the 15 neurons result in many pair-wise spike time relationships between neurons, which we show schematically in Figure 2*D*. These pair-wise relationships can also be visualized as cross-correlograms for all neurons, which we show in Figure 2*E*.

The profiles in Figure 2*A–C* are directly comparable to the three profiles of spike timing networks extracted using our approach (for how to interpret the profiles, see Materials and Methods, Extracting spiking timing networks from neuronal spike recordings), and are used below (Recovery of simulated spike timing networks with spiking jitter when surrounded by spiking noise, Recovery of simulated spike timing networks with partial spiking when surrounded by spiking noise, and Cross spectra normalization diminishes effects of differential firing rates of units and trials, for judging recovery of the simulated networks by the extracted spike timing networks). Note, the absolute values of the neuron and trial profiles are not meaningful, only the within-network ratios are (see Materials and Methods, SPACE describes time consistency-induced phase coupling in cross spectra). As such, it is not the spike sequence repeats per trial that is described by the trial profile (i.e., 0, 1, 2, 3; Fig. 2*C*), but rather the ratio between them (e.g., a trial with three sequences having a weight three time that of a one-sequence trial).

To investigate when the recovery of the simulated networks fails, we varied the strength of three kinds of noise (see Materials and Methods, Simulating and extracting noisy spike timing networks). These were: (1) spiking noise, or non-network spikes, superimposed on the spike sequences (Fig. 2*F*); (2) jitter of each spike in a spike sequence occurrence; and (3) partiality of network spike sequences (random spikes missing from the sequence). The range of each of the noise levels was chosen to provide an intuition for when an expected network can still be recovered, and to progressively result in failure to recover the simulated networks. As such, the higher levels are not necessarily physiologically reasonable. The simulated networks were also different in size, spike sequence timing, spatial overlap, and trial overlap, to increase the likelihood that any related weaknesses of the technique would be revealed.

### Recovery of simulated spike timing networks with spiking jitter when surrounded by spiking noise

**Figure 3.**
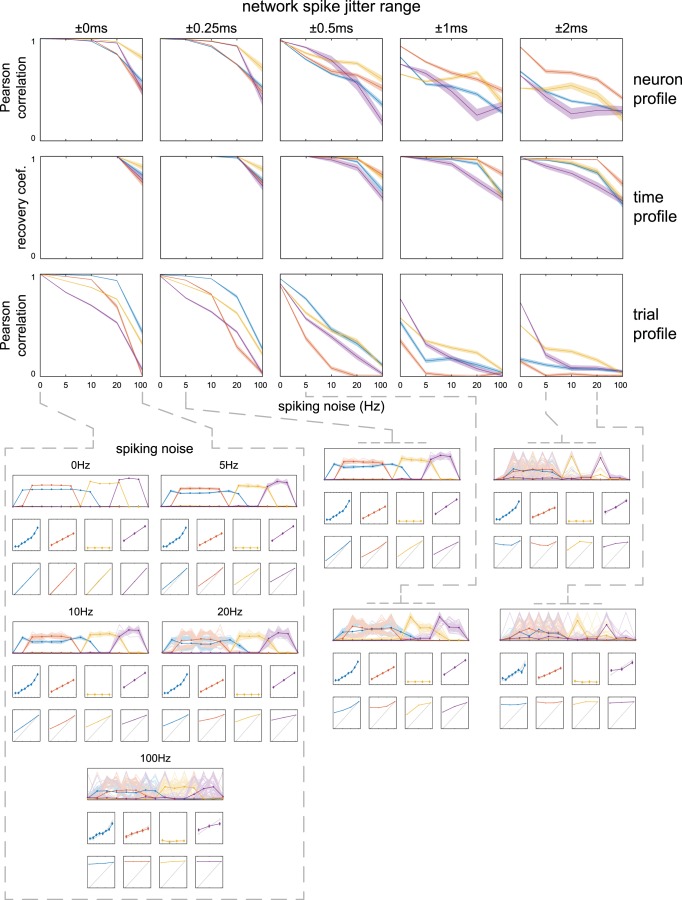
Recovery of simulated spike timing networks with spiking jitter and spiking noise. Networks were simulated 50 times at five levels of spiking jitter and five levels of spiking noise. Recovery of the neuron and trial profiles are shown as Pearson correlations between the extracted and simulated networks (ranged from -1 to 1, visualized from 0 to 1; averaged over simulations; shading = SEM). The recovery of the time profile is shown by a recovery coefficient ranging from 0 to 1 (perfect recovery; averaged over simulations; shading = SEM). Networks are colored as in Figure 2. Bottom panels visualize extracted networks as in Figure 2 at several example jitter and noise levels. Neuron profiles are shown as means over simulations (shading = SD), with that of individual simulations as thin lines. Time profiles are displayed as average over simulations (error bar = SD; aligned using average difference between simulated and recovered networks). Trial profiles show means over trial weights per number of simulated sequence repeats, averaged over simulations (error bar = SD). The simulated trial profiles and time profiles are indicated in gray for reference. Note that (1) when spiking noise and jitter increased, the trial profiles baseline (weights of noncontributing trials that should be 0) gradually increased; (2) spiking noise had a stronger effect on the trial profiles of networks with fewer neurons; and (3) the time profiles were more robust to noise than the neuron profiles and trial profiles, with accurate recovery even when spike jitter was a multiple of the between-neuron time delays. Also note in the examples that as noise increased, matching of simulated networks to extracted networks became troublesome, leading to differences between network-specific recovery becoming less meaningful. See Materials and Methods, sections Simulating and extracting noisy spike timing networks and Quantifying recovery of simulated spike timing networks.

Neurons can be noisy, and any spike sequence of a spike timing network is likely embedded in other spikes of the same neurons. Furthermore, precise spike times depend on the fluctuating membrane potential of the neuron and other factors, potentially adding temporal jitter. To investigate how these two factors influence network characterization, we simulated spike timing networks with different levels of background spiking noise and with different levels of jitter of each spike in the spike sequences. Networks were simulated 50 times for each combination of the noise factors. We computed recovery of simulated networks and show the result in Figure 3. Recovery of the neuron and trial profiles was computed as the Pearson correlation between the recovered and simulated profiles. For the trial profiles, the correlation also directly reflects the recovery of the linear modulation of network activity across the task (perfect recovery is 1). Recovery of the time profiles was computed using a recovery coefficient that ranges from 0 to 1 (perfect recovery; see Materials and Methods, SPACE describes time consistency-induced phase coupling in cross spectra).

Firstly, we observe that with reasonable jitter (i.e., ±0.25 ms compared to 0 to 2.5 ms spike sequence delays) and spiking noise (e.g., 20 vs 1.2 Hz average network spiking rate) the neuron and time profiles were recovered with reasonable accuracy, with the trial profile being the most affected. At 20 Hz spiking noise and ±0.25 ms jitter the linear modulation of network activity was still visible but weakened [Fig. 3, bottom: mean (SEM) over simulations of Pearson’s correlations for network 1-4: 0.78 (0.02), 0.29 (0.02), 0.62 (0.01), and 0.44 (0.01)]. Shown in the examples (Fig. 3, bottom), the effect of noise on the trial profile can be observed as a shrinking of the ratios between loadings of trials with a different number of simulated network sequences and an increase in the trial profiles “baseline”; the loadings of those trials which had zero network sequences. The latter is important in practice, because under the assumption that a network is not active in all trials, the lowest trial loadings with respect to the higher trial loadings can be used as an indication of the reliability of network parameters. Secondly, we observe that, except from the largest jitter case (±2 ms), network spike jitter had a similar effect on recovery of network parameters as spiking noise, as evidenced by the similarity between the 10 Hz/0 ms and the 5 Hz/±0.25 ms cases, and the 20 Hz/0 ms and the 5 Hz/±0.5 ms cases. Thirdly, we observe that, under strong noise conditions (>20 Hz spiking noise and >±1 ms jitter), the linear modulation of network activity became very weak to largely invisible (maximum mean Pearson’s correlation over simulations of 0.11, 0.04, 0.23, and 0.08 for network 1-4). Regarding network specific recovery, although there was some variation in recovery, apart from the above, the differences were minimal and did not highlight a sensitivity to a particular aspect of the simulated networks.

### Recovery of simulated spike timing networks with partial spiking when surrounded by spiking noise

**Figure 4.**
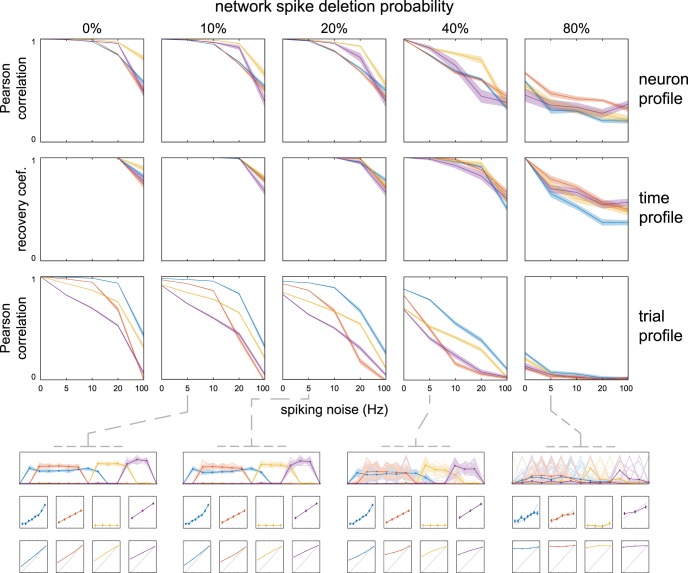
Recovery of simulated spike timing networks with partial network spiking and spiking noise. Networks were simulated 50 times at five probability levels of spike deletion, and five levels of spiking noise. Probability is the chance for each individual (non-noise) spike to be deleted. Recovery and examples are displayed identically to Figure 3. Note that (1) the effect of spike deletion affected the neuron profiles, time profiles, and trial profiles similarly to that of spike jitter and spiking noise; and (2) even when the spiking sequences of the networks were highly variable (80% chance of each spike’s absence) the networks could still be identified in the examples at 5 Hz spiking noise. See Materials and Methods, sections Simulating and extracting noisy spike timing networks and Quantifying recovery of simulated spike timing networks.

To investigate how partial spiking in spike timing networks, i.e., not all member neurons joining in each spike sequence, affects characterization of the full spike sequences, we simulated networks where each spike of a sequence had a chance to be deleted. Similar to the above, we did so 50 times for each level of spike deletion probability, and of spiking noise. The results are shown in Figure 4. We observe that (1) as the chance of spike deletion increased, recovery accuracy was decreased; (2) as with spiking jitter/noise, the trial profile was more affected by noise than the neuron profile; (3) as with spiking jitter/noise, the effects of spike deletion on recovery were similar to those of spiking noise; (4) the full spike sequences in the time profile were accurately extracted under reasonable noise (20 Hz) with 40% probability of spike deletion, although the majority of individual spike sequences were incomplete; and (5) under the same noise conditions the linear modulation of network activity was weak but detectable for networks 1 and 3, and nearly invisible for networks 2 and 4 [mean (SEM) Pearson’s correlation over simulations of 0.39 (0.02), 0.06 (0.02), 0.29 (0.02), and 0.08 (0.02) for networks 1-4]. These differences possibly stem from network overlap (network 2 shares all its neurons with network 1) and network size (network 3 is the smallest).

### Cross spectra normalization diminishes effects of differential firing rates of units and trials

The spike timing networks simulated above were extracted under noise related firing rates that were identical over neurons and over trials. This was chosen to show the overall effect of spiking noise but is atypical for real recordings. Here, we show the effect on network recovery of firing rate differences between neurons and trials, while keeping the number of spike sequences constant.

**Figure 5.**
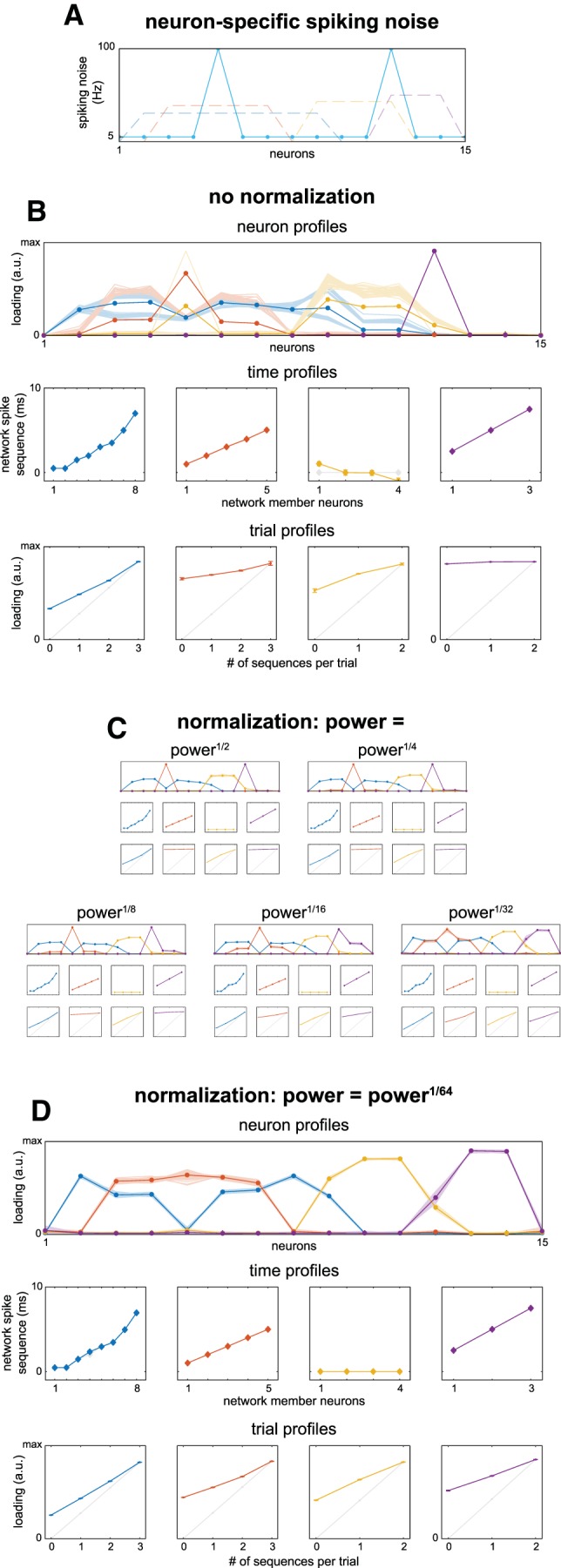
Cross spectra normalization diminishes effects of differential neuron firing rates. In realistic neuron recordings, the firing rate of neurons typically differ. To show the effect of differential firing rates on network recovery, we simulated spike timing networks (spiking jitter = ±0.25 ms; spike deletio*n* = 10%) 50 times with two neurons having 100 Hz spiking noise, the other neurons 5 Hz. To improve network recovery, the cross spectra can be normalized. One method is to normalize them such that the power of the cross spectra becomes equal to their *N*th root. ***A***, Spiking noise as a function of neurons, with the simulated neuron profiles in the background. ***B***, Network recovery without normalization. Although the networks are recognizable, recovery was clearly affected. Networks are displayed identically to examples in Figure 3. ***C***, The effect of square, 4th, 8th, 16th, and 32nd root power normalization on recovered networks, culminating in (***D***) recovered networks after 64th root power normalization. See Materials and Methods, sections Normalizations of the cross spectra and Simulating and extracting noisy spike timing networks.

We first show the recovery of simulated networks when the firing rate differs over neurons (Fig. 5). We simulated networks 50 times, with neuron 5 (a member in networks 1 and 2) and neuron 12 (member in networks 3 and 4) having 100 Hz spiking noise, the other neurons 5 Hz (Fig. 5*A*). Network spiking jitter was ±0.25 ms. The recovery of the networks (Fig. 5*B*, compare to Fig. 2) was distorted: (1) the neuron profiles of the networks for neurons 5 and 12 were strongly increased/decreased; (2) the noise of neuron 5 led to a strong loading for network 3, of which it was not a member; (3) network 4 was dominated by neuron 12; (4) the trial profiles showed decreased recovery (compared to Fig. 2); and (5) although the time profile of networks 1, 2, and 4 were not (noticeably) distorted, network 3’s is. Overall, the differential firing rate can be said to have pulled the estimated network parameters toward those neurons with more spiking. This effect however, can be substantially reduced by normalizing cross spectra before network extraction. Here, we show the effect of normalizing cross spectra such their power is equal to their *N*th root (see Materials and Methods, Normalizations of the cross spectra), reducing differences in firing rates. We show its effects progressively by using *N* = 2, 4, 8, 16, 32 (Fig. 5*C*), and ending with *N* = 64 (Fig. 5*D*). We observe that (1) the recovery of the neuron profiles was improved, with network 1 showing the most remaining distortion at neuron 5; (2) the trial profiles were similar to the case with 5 Hz spiking noise for all neurons (Fig. 2); and (3) the recovery of the time profile of network 3 was improved such that the distortion is minimal.

**Figure 6.**
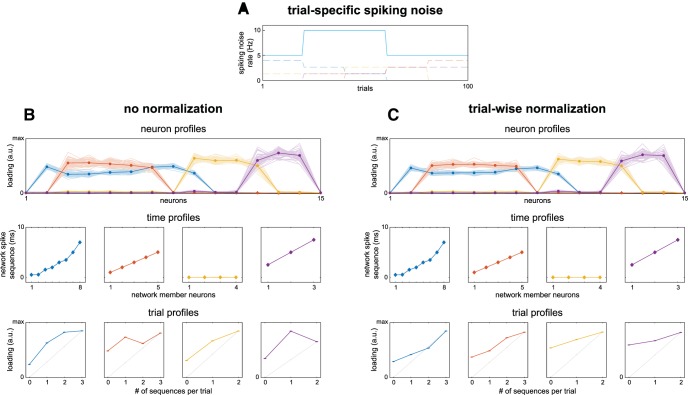
Trial-wise cross spectra normalization diminishes effects of differential trial firing rates. In realistic recordings, the firing rate of neurons can differ over trials. To show its effect on network recovery, we simulated spike timing networks (spiking jitter = ±0.25 ms; spike deletion = 10%) 50 times with 40 trials having 10 Hz spiking noise (for all neurons), the other trials 5 Hz. To improve network recovery, the cross spectra can be normalized in a similar way as for differential neuron firing rates. Here, we normalize the cross spectra of each trial such that their power is equal to that summed over trials. ***A***, Spiking noise as a function of trials, with the simulated trial profiles in the background. ***B***, Recovered networks with trial-wise normalization. Networks are displayed identically to examples in Figure 3. The trial profiles of the recovered networks were strongly affected. ***C***, Like ***B*** but for networks recovered after trial-wise normalization. While the trial profiles still deviated from the simulated networks, the ratios of their weights w.r.t. the number of simulated sequence repeats were partially restored. See Materials and Methods, sections Normalizations of the cross spectra and Simulating and extracting noisy spike timing networks.

To investigate the effect of differential spiking rate over trials, we simulated networks with 5 Hz spiking noise, except for trials 21–60, which had 10 Hz spiking noise (Fig. 6*A*). Network spiking jitter was set at ±0.25 ms spiking jitter, and networks were simulated 50 times. The trials with additional spiking noise were chosen such that they both involved 100% of trials of sequence repeats (1× and 2× for network 1, 1× for network 2, 1× for network 4) and a partial set of sequence repeats (50% of 1× and 2× for network 3, 50% of 0× for network 2). The recovery without normalization is shown in Figure 6*B*. We observe that (1) the recovery of the neuron profiles and time profiles was similar to the case of 5 Hz noise and ±0.25 ms spiking jitter (Fig. 2), and as such, they were minimally affected by the differential noise over trials; (2) the linear modulation of network activity was recoverable, but weakened, for all networks [especially network 3; mean (SEM) Pearson’s correlation over simulations of 0.93 (<0.01), 0.67 (0.01), 0.76 (<0.01), 0.36 (0.01) for network 1-4] compared to without trial variations of firing rate [0.98 (<0.01), 0.94 (<0.01), 0.89 (<0.01), 0.77 (0.01); Fig. 3]; and (3) the trial profile loadings for those trials affected by increased spiking noise were distorted such that the ratios of loadings no longer reflected the correct order of the number of sequence repeats (i.e., 1× > 2× trials for network 2 and 4). Although the linear modulation was moderately recoverable, the latter means an investigation of the network activities in specific trials of network 2 and 4 (supported by, e.g., an independent samples *t* test) would have resulted in the incorrect conclusion of more network activity being present in 1× compared to 2× trials. As was the case for differential noise over neurons, normalization of the affected dimension can improve recovery. Here, we normalized the cross spectra such that their power for every trial is equal to their power summed over trials (see Materials and Methods, Normalizations of the cross spectra). Crucially, this does not affect the ratio of the off-diagonal elements to the diagonal (power). As such, trials that have many spike sequences (strong off-diagonal elements compared to power) are still distinguishable from trials with few spike sequences (weak off-diagonal elements compared to power). Note, as well, that this trial-wise normalization is unrelated to the neuron-wise normalization in the above, and they can be applied jointly. We show the result of the trial-wise normalization in Figure 6*C*. We observe that (1) the trial profile recovery was improved such that the order of their loadings again reflected the order of the number of sequence repeats; (2) recovery of the linear modulation of network activity was greatly improved [mean (SEM) Pearson’s correlation over simulations of 0.96 (<0.01), 0.96 (<0.01), 0.82 (<0.01), 0.89 (<0.01)]; and (3) although improved, the trial profiles’ recovery was poorer than those at equal noise levels for all trials (Fig. 3). We additionally observe that the normalization also affected the trial profile loadings of trials that did not have increased noise. This was most noticeable in the loadings for those trials of network 4 that had zero and two sequence repeats (trials 1–20 and 61–100): the ratio of the loading of zero 0 repeats to that of two repeats was much higher without normalization (Fig. 6*B*), than with normalization (Fig. 6*C*). As trials with zero repeats should ideally have a loading of 0, the higher this ratio the better. Interestingly, although the trial profile showed worse recovery overall, the recovery of the linear modulation of network activity after trial-wise normalization was better than the recovery at equal noise levels across trials without normalization, especially for network 4 [mean (SEM) correlation of 0.89 (<0.01) and 0.77 (0.01), respectively]. This was likely caused by the trial profiles of the former showing less variability than those of the latter (i.e., the coefficient of variation of the trial profile of network 4, averaged over simulations, was 14.1% and 27.9%, respectively).

### Spike timing networks extracted from real recordings reflect between-neuron spike timing relationships

**Figure 7.**
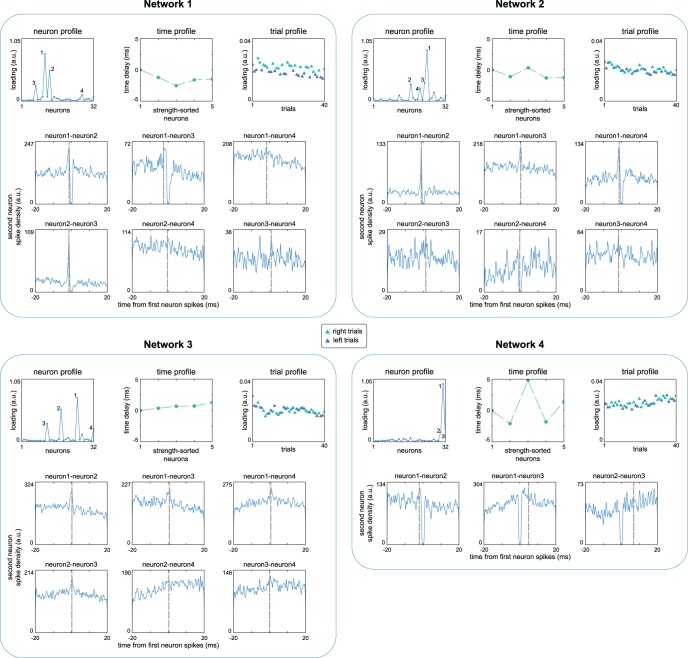
Example spike timing networks extracted from rat medial prefrontal cortex and hippocampus. We extracted four spike timing networks from recordings in which a rat either had to take the left or right arm of a figure-eight T-maze. The number of networks to extract was estimated using a split-half approach. The first row of each network shows the neuron profile, the time profile, and the trial profile. The time profile only shows the strongest five neurons of the trial profile (as given by the neuron profile). Several of the strongest neurons are highlighted in each neuron profile. To show that the networks reflect spike timing consistencies in the data, we also show cross-correlograms in the second and third row. The cross-correlograms of each pair of the highlighted neurons are shown as spike densities, the *y*-axis limit roughly reflects spike counts. The dashed gray line is the time delay between the neurons as given by the time profile of the network. We observe the following. For network 1, neuron pairs 1-2, 1-3, and 2-3, the extracted time delays are close to the cross-correlogram. Although the 4th neuron has a higher weight than the nonhighlighted neurons in the neuron profile, the cross-correlograms are not as strongly peaked as for the other pairs. For network 2, the extracted time delays of pairs 1-2, 1-3, and 1-4 are closest to their cross-correlograms. Although for network 3 the cross-correlograms show weaker spike timing consistency (higher baseline spike density), the extracted time delays of pair 1-2, 1-3, 1-4, and 2-3 are close to their peaks. Network 4 involves few strong neurons, as indicated by the neuron profile; only the neuron pair 1-2 is close to its peak. See Materials and Methods, Extracting spike timing networks from recordings of rat medial prefrontal cortex and hippocampus.

To provide a proof-of-principle we extracted spike timing networks extracted from spike recordings from medial prefrontal cortex and hippocampus of a rat performing an odor-based delayed matching-to-sample task (Fig. 7; see Materials and Methods, Extracting spike timing networks from recordings of rat medial prefrontal cortex and hippocampus). After odor presentation, the rat had to run through the left or right arm of a figure-eight T-maze to obtain its reward. Networks were extracted similarly to the simulations above, using a neuron-wise 32nd root power normalization, and a split-half reliability approach to determine the number of networks (see Materials and Methods, SPACE describes time consistency-induced phase coupling in cross spectra). This resulted in four extracted networks.

We show neuron profiles, time profiles, and trial profiles for each extracted spike timing network in Figure 7. To provide a ground-truth estimate of whether the between-neuron spike times from the networks reflect real spike timing relationships in the recordings, we also show for each network continuous cross-correlograms (computed post-hoc; see Materials and Methods, Extracting spike timing networks from recordings of rat medial prefrontal cortex and hippocampus) of the neurons mostly strongly contributing to each network. Importantly, in each of these cross-correlograms we indicate when the cross-correlation is expected to be highest, based on the time profile of the networks.

For network 1, neuron pairs 1-2, 1-3, and 2-3 had peaks in their cross-correlograms that matched the time profile’s spike timing relationships within 0.03, 0.03, and 0.06 ms, respectively. Neuron 4 does not appear to have consistent spike timing relationship with the first three, which is unsurprising given that its weight in the neuron profile is much weaker (suggesting its weight reflects, at least mostly, noise). Although there appears to be a difference in network activity between left and right trials, this likely due to firing rate differences between conditions, as trial profiles calculated on trial-wise normalized cross spectra showed no statistically significant difference (see Materials and Methods. Normalizations of the cross spectra; this should be interpreted with caution however, as the profile’s lowest weights suggested they remained noisy). For network 2, neuron pairs 1-2, 1-3, and 1-4 had cross-correlogram peaks that matched the time profile within 0.09, 0.07, and 0.19 ms, respectively. Neuron pair 2-3 and pair 3-4 did not have single cross-correlogram peak (although their center peaks matched within 0.17 and 0.12 ms, respectively), and neuron pair 2-4 appears inhibitory. These observations could indicate that the spike sequence did not involve all four neurons in a subset of trials. It is useful to reiterate here, that the extracted spike sequence should be considered only at the level of the full recording (i.e., cross spectra of all trials). That is, the extracted spike sequence should be considered as a description of the *N*-way relationship between *N* neurons, i.e., the largest possible spike sequence for the network, and serve as a starting point for targeted analyses. Network 3 show a similar pattern as network 1 and 2 according the cross-correlograms, with the peaks of neuron pairs 1-2, 1-3, 1-4, and 2-3, matching the time profile within 0.10, 0.09, 0.05, and 0.19 ms, respectively. Network 4 likely reflects consistent spike timing only between the strongest two neurons (matching within 0.08 ms), as the neuron profile has few neurons with strong loadings.

## Discussion

Identifying and investigating cell-assemblies with spike timing consistency between neurons is key to gain a further understanding of their role in neuronal coding (Bienenstock, 1995; Singer, 1999; Tiesinga et al., 2008; Panzeri et al., 2010), but finding them is a tremendous challenge due to the possible complexity of patterns of between-neuron spike time delays. Here, we introduced and validated, in simulated and real data, a novel approach for extracting networks defined by their between-neuron spike timing consistency, when forming sequences of time-shifted spikes, from neuronal spike recordings (for other types of interactions, see Lindemann et al., 2001). The key features of this approach are that (1) networks and their spike sequences can be extracted regardless of their complexity in size and spike timing patterns, and (2) the spike sequences of the networks are specified with high temporal precision. Networks consist of three profiles, describing (1) which neurons are involved in which networks, (2) with which spike timing pattern, and (3) in which trials or conditions. The latter can in principle be used as an index for network activity. Together, these profiles form a parsimonious description of the spike timing patterns in the recording and can used as a basis for subsequent spike train analyses of experimentally relevant variations in network subsets. Using simulations, we showed how the extracted networks were affected by spiking jitter, variability in network participation by its member neurons, and non-network related spiking activity. Networks were recoverable under reasonable noise conditions, with the time profile being especially robust to the simulated noise. Although the trial profiles were strongly influenced by noise, they still tracked simulated network activity to a degree. Using neuronal spike recordings from rats, we showed we were able to extract networks from real recordings, of which the time profile reflected between-neuron spike timing consistency that matched cross-correlograms with high accuracy. Together, this shows that our approach can be useful for the investigation of spike timing networks.

The extracted networks can be of arbitrary complexity in size and time delays. This is a consequence of the fact that the underlying method finds networks not in the neuron-by-time time series, but in the neuron-by-neuron cross spectra. These cross spectra contain all of the spike timing consistencies of the spike sequences, condensed into between-neuron phase coupling. Networks can be separated when their spike sequences have different between-neuron phase coupling patterns, and differences in phase coupling patterns over trials (or epochs) increases their separability. Networks are extracted by finding those neuron, time, and trial profiles whose phase coupling patterns explain the most variance in the cross spectra. Because the estimated profiles have the same size for each network, larger networks only differ from smaller networks by their different distribution of weight magnitudes. As a larger network does not involve estimating a larger number of weights, there is no combinatorial explosion with increasing network size. In fact, higher complexity networks are likely easier to find than lower complexity networks, as they will typically explain more variance in the cross spectra. The above is different from techniques that search for template spike sequences in their original neuron-by-time representations (Abeles and Gerstein, 1988; Nádasdy et al., 1999; Tetko and Villa, 2001; Lee and Wilson, 2002; Schnitzer and Meister, 2003; Ikegaya et al., 2004; Gansel and Singer, 2012). As these search for exact spiking templates, they have to do so within some restricted space to avoid a combinatorial explosion. Although finding high complexity networks is impractical with such approaches, they have the advantage of being able to find spike sequences that repeat very few times in the course of a recording. Because our approach is most sensitive to those networks that explain the most variance in the cross spectra, it is not well suited for finding sequences with very few repeats, as they typically explain very little variance in the cross spectra. As such, our approach trades sensitivity to such sequences for sensitivity to sequences with arbitrarily high complexity but that are more prominent.

An important aspect of the method behind our approach is that it is a decomposition of between-neuron cross spectra over frequencies and trials into sets of network profiles. Because this decomposition attempts to find profiles that parsimoniously explain all of the variance in the cross spectra, its profiles need to not only describe between-neuron spike pairs, but also their total number of spikes. Importantly, the latter typically outnumber the former to a strong degree (Gochin et al., 1991; Nelson et al., 1992; Kreiter and Singer, 1996; Brosch and Schreiner, 1999). This impacts the interpretation of the neuron profile weights. For any two neurons, their weights in the neuron profile need to describe four magnitudes of the cross spectra: their total number of spikes in the magnitude of their cross spectral power, and their spike timing consistency in the magnitude of their off-diagonal elements. In the case these magnitudes differ, the neuron profile weights become a compromise, and are drawn to those magnitudes that explain the most variance. These weights should therefore be interpreted with caution and should be considered more as an indication of network membership when sufficiently away from 0, than as a straightforward index into the strength of their spike timing consistency. This is also the reason why a neuron-wise normalization of the cross spectra is advisable, as it will reduce the effect of firing rate differences. In fact, in our experience, when the cross spectra are not neuron-wise normalized, few extracted networks will consist of more than one neuron (i.e., artefactual networks that are not based on spike timing). If it is also the case that the total number of spikes of neurons differs more over trials than the number of their spike pairs do, then the trial profile weights will be drawn toward the former, as they will explain more variance in the cross spectra. This was likely the case for the networks we extracted from rat hippocampus and medial prefrontal cortex (Fig. 7, network 1), and is also likely the reason why the trial profile was strongly impacted by simulated spiking noise. A trial-wise normalization for this was introduced, that in the specific case of our simulations, improved recovery of the linear modulation of network activity. Nonetheless, the trial profile remained sensitive to noise and, as such, should be used with caution, ideally with complementary analyses (such as a targeted search, see below). The above contrasts with a previous application on human electrophysiological recordings, where the trial profile was less sensitive to noise (likely caused by more spatially extended networks; van der Meij et al., 2015, 2016).

Our approach describes the structure of spike timing consistencies in the cross spectra of the entire recording. This means that the spike sequences represented by each network’s time profile describe the spike timing consistencies of the involved neurons over the entire recording. As such, the time profile reflects an aggregate spike sequence, one that does not necessarily exactly repeat in each of the involved trials. For example, some trials might only contain a part of the sequence. This property can also be considered beneficial, and it is something we explicitly tested in our simulations with partial spike sequences. In the case of strong variability in the exact spike sequence of every trial, the “main” sequence could still be identified.

The aggregate nature of our spike sequences contrasts with those of approaches that search for exactly repeating spike sequences (Abeles and Gerstein, 1988; Nádasdy et al., 1999; Tetko and Villa, 2001; Lee and Wilson, 2002; Schnitzer and Meister, 2003; Ikegaya et al., 2004; Gansel and Singer, 2012). These approaches typically also incorporate some form of statistical testing of the identified spike sequences, which is necessary to obtain more certainty that the found sequences are not an accidental consequence of statistical properties of the firing rates (for a discussion of surrogate data for this purpose, see Grün, 2009). Importantly, we consider our approach not as an alternative to the above, but as complementary. That is, our spike timing network profiles can be used to construct a network-specific spiking template with between-spike time delays at high temporal resolution, that can be used in approaches like the above to locate discrete occurrences of the network’s spike sequences. This would allow for subsequent investigations into, e.g., spike time variability within sequences, variable occurrence of sequences over conditions, and spike sequence completeness, of network spike sequences with a complexity that would otherwise be prohibitive.

Arguably the approaches closest to ours are those that also depend on neuron-by-neuron representations to investigate spike timing consistency. Of these approaches, some start from a principal component analysis (PCA) on the between-neuron cross-correlations (Chapin and Nicolelis, 1999; Peyrache et al., 2010; Lopes-dos-Santos et al., 2011). These approaches result in a neuron profile per component, describing correlated and anti-correlated neurons, and a temporal profile, providing a component activity time course of some form that can be matched to the original neuronal spiking time series. The biggest difference to our approach, is that, in those methods, between-neuron timing information is lost when transforming the neuronal spiking time series to cross-correlation matrices. Apart from losing the specification of the order and timing of the network spiking sequence, this also makes it more difficult to distinguish between those networks that involve the same neurons, but at different between-neuron spike times. This adds unto the rotational ambiguity of PCA that influences network identification and separation, although Lopes-dos-Santos et al. (2011) made significant advances with respect to the latter. In comparison, the method behind our approach (van der Meij et al., 2015), and related methods (Harshman and Lundy, 1994; Bro, 1998; Kiers et al., 1999; Sidiropoulos et al., 2000; Mørup et al., 2008), extracts networks that are unique without rotational ambiguity, and separates them on the basis of their different structure across neurons, frequencies, and trials. Several other approaches use cross-correlation matrices in way that did allow for an investigation of between-neuron spiking at time delays (Schneider et al., 2006; Nikolíc, 2007; Humphries, 2011), but these approaches were not targeted at identifying and separating networks and their spiking sequences.

In summary, we have presented an approach that can extract networks defined by their between-neuron spike timing consistency, with arbitrary network size and high temporal precision of the identified spike sequences. Especially the latter is important considering the growing number of neurons that can be recorded simultaneously, and the complexity of spike sequences that can thus be measured. Ultimately, the usefulness of our approach and those related to it, lies in whether spike timing plays a crucial role in large, distributed, neuronal networks. Being able to search for these networks with increased sensitivity is essential to the investigation of their existence and function.

10.1523/ENEURO.0379-17.2018.ed1 Extended Data Supplementary Analysis Software. Download Extended Data, ZIP file.

## Synthesis

Reviewing Editor: Marlene Bartos, University of Freiburg Decisions are customarily a result of the Reviewing Editor and the peer reviewers coming together and discussing their recommendations until a consensus is reached. When revisions are invited, a fact-based synthesis statement explaining their decision and outlining what is needed to prepare a revision will be listed below. The following reviewer(s) agreed to reveal their identity: Eelke Spaak

The manuscript was heavily disused among two reviewers and the reviewing editor. One main problem was addressed regarding the ‘novelty’ of the method proposed in the study, in particular “the adaption to the spike train case”. The novelty aspect does not really stick out well. The authors should present it much more in that way. You will see below that several detailed questions have been addressed to details of the model. It would be important to address them. Some of the changes will require mild changes to the text but others may require additional modifications of the model.

The main problems must be addressed which are as follows:

1. Include the fact that sometimes neurons don't fire in the equations. This seems esp. a problem given that the authors look at sequences on a very fine time-scale, with short wavelets. For many problems - like sequences in sharp wave ripples - or latency coding after stimulation onset, fast-time scale sequences are important.

2. The normalization for rate with the power scaling seems rather ad-hoc and arbitrary, and has an optimization issue for several components (see below) and the problem with zeros. Please address these issues.

3. (1) While reading the paper, initially I was very excited about the possibility to compare trial weighting scores of different networks in real experimental settings, in order to determine differential engagement of network motifs with experimental tasks, over and above any differences in firing rates. The authors hint several times that this could be an advantage of their approach (e.g. lines 45, 98-99, 109-110, 178), and I agree that this aspect is one of the most promising of their approach over other techniques. However, they never quantify explicitly how this would work (a statistical test?). They do, however, hint that they tried something like this on the real data from Fujisawa et al. (2008), and although there was a difference between trial types (Figure 7; network 1), this was ‘likely caused by firing rate differences’ (line 655). I would encourage the authors to explicitly quantify the sensitivity and specificity of their technique for detecting *differences* in connectivity patterns between different epochs of a task, given potential firing rate confounds. Of course, the changes in estimated trial weightings (especially when adding the trial-specific noise as in Figure 6) go into this question to some extent, but the treatment by the authors so far remains qualitative.

4. Related to this, the main conclusion of Fujisawa et al. (2008) was that there *are* differences in connectivity between two trial types. Do the authors of the present manuscript believe that this conclusion was still confounded by firing rates, and that properly controlling for this confound invalidates the conclusion (a very real possibility)?

5. Also related, I would like to see some more explicit statements on what the authors mean when they say that their technique provides a ‘starting point’ for further analyses (e.g. lines 44, 767-768). Wouldn't ‘zooming in’ by this technique, and then subsequently employing a statistical test only on the zoomed-in networks, invalidate the latter test (i.e. ‘double dipping’)?

6. I would like to see some simulations of no network structure at all, i.e. only uncorrelated spike trains. Does the split-half reliability approach of network number correctly come up with N = 0 in those cases?

7. For the application to real data, there are some zero-valued dips in the cross-correlograms. Are these caused by spike sorting units within the same contact? In general, please specify in the methods whether units all came from different contacts or also the same, and (roughly) how spike sorting was done, as this can interact with the detection of connected networks.

1) Linear phase assumption. The first assumption that is embedded in the model is that the phase is a linear function of frequency.

“The crucial principle here, is that time differences in the time domain

translate to phase differences in the frequency domain, linearly increasing with frequency” (p. 153).

8. This is true in only a limiting cases - where one time series is a copy of the other - but the phase spectrum generally can have a more complex dependence on frequencies

(see Lindemann et al., Journal of Neuroscience Methods, 2001).

The simulations here are set up to create such a limited case.

Doubts thus remain about the general applicability of the SPACE model to spiking networks.

9. Component estimation: splitting the recordings in half, with odd and even numbered spikes, seems a rather odd procedure to determine the reliable components.

Can we generally assume that the odd and even numbered spikes have the same sequence behavior? Furthermore, do we not throw away sequences in which both odd and even spikes constitute the sequence? How do you deal with sequences that can be carried by one spike in one neuron (this happens in hippocampus for example).

10. The authors say they use a measure of similarity between the network component derived from the odd and even half of the spikes, and then accept the component

if the network is reliable enough (and then estimate more components). It is unclear to me whether there is any theoretical guarantee that this works in practice and the cut-off value for similarity is not provided by the authors. In fact in the section about rate normalization the authors say that “This has the unintended consequence however, of prematurely stopping a split reliability estimation of the number of networks to extract. This is most likely due to power normalizations increasing the variability of the order in which networks are extracted, causing different networks to be extracted from the different splits when extracting less than the optimal number of networks.” That does not give the reader much confidence about finding components with the split half procedure.

11. It is completely unclear to me how the method deals with zeros - neurons that do not spike in some trial, or given the split half procedure, neurons that fire one spike.

Given lognormally distributed firing rates, one expects that in many practical cases, at least a subset of neurons do not fire in some trials. The equations, as they stand, contains divisions by 0, for the normalizations.

12. The relationship between the multiple components remains unclear to me from the description of the method here. How does the method deal with distinct sequences that are highly correlated? Will they end up in one component? How many components can the method theoretically distinguish, for n neurons? Does it end up with a few components, like PCA, in practice? All of this is extremely important but not discussed in the text.

13. The authors propose neuron-wise normalization as a necessary tool when neurons have different rates. The consequence of that is, according to the authors, that the weighting becomes dominated by one neuron. However the construction of this method, p. 12, is cumbersome. There is some power factor 1/N by which the power is scaled, and apparently the power cannot be equalized (N cannot be too large) because then the optimization fails, according to the authors. The authors say that N has to be determined with trial and error, but the criterion for determining an optimal N seems subjective and arbitrary. Also the authors say that it is recommended to first find networks without trial normalization, because again there are apparently optimization problems. Furthermore it is unclear how the method deals with zeros, that is neurons that do not fire in some trials. The equation of trial normalization right now cannot deal with zeros (and ones, given the split half procedure).

14. The time length of the wavelets seems also an arbitrary variable. The authors say that there is some optimum for the length of the wavelets. Too short wavelet makes one blind to longer sequences, but too long wavelets diminish sensitivity because there are too many spike pairs going into the cross-spectrum. In the simulations, the authors know what they are looking for, but in practice, how does one determine the wavelet length?

15. Minor points:

- Line 107: describe -> describes

- Lines 279-281: sentence is somewhat unclear.

- Line 336: the number of frequencies required in the original (non-spiking) formulation of the method seems irrelevant to me for the present application to spiking networks (although I might be missing something).

- Lines 437-440: unclear sentence; also I don't see how mere *addition* of spike trains results in a cross-correlogram.

- Line 743: differ -> differs

-Page 6, equation ?. j_1, j_2, what is the interval for the indices? Are the variables optimized for each pair, or is j_1 also combined with j_3? This is unclear from the equation and text.

-In general, indicating whether variables are a function of time or frequency would make the math a lot more readable.

- Line 349 - the variable Ydiag is not defined.

- f might be a more logical choice for frequency, k for factor.

- how do you deal with the edges with the convolution, p. 10

-Many of the figures are barely visible, both printed and on screen.
